# Explainable Machine Learning Reveals Time-Dependent Cognitive Risk in Minor Neurocognitive Disorder: Implications for Health Promotion and Early Risk Stratification

**DOI:** 10.3390/biomedicines14040880

**Published:** 2026-04-12

**Authors:** Anna Tsiakiri, Christos Kokkotis, Dimitrios Tsiptsios, Leonidas Panos, Nikolaos Aggelousis, Konstantinos Vadikolias, Foteini Christidi

**Affiliations:** 1 Department of Neurology, Medical School, Democritus University of Thrace, 68100 Alexandroupolis, Greece; atsiakir@med.duth.gr (A.T.); lpanos@med.duth.gr (L.P.); kvadikol@med.duth.gr (K.V.); 2 Department of Occupational Therapy, School of Physical Education, Sport Science and Occupational Therapy, Democritus University of Thrace, 69100 Komotini, Greece; ckokkoti@affil.duth.gr; 3 3rd Department of Neurology, Aristotle University of Thessaloniki, 54124 Thessaloniki, Greece; tsiptsios.dimitrios@yahoo.gr; 4 Department of Physical Education and Sport Science, School of Physical Education, Sport Science and Occupational Therapy, Democritus University of Thrace, 69100 Komotini, Greece; nagelous@phyed.duth.gr; 5 Department of Psychology, School of Philosophy, National and Kapodistrian University of Athens, 15784 Athens, Greece

**Keywords:** minor neurocognitive disorder, health promotion, explainable machine learning, diagnostic prediction, cognitive risk

## Abstract

**Background/Objectives**: Minor neurocognitive disorder (minor NCD) represents a heterogeneous and potentially modifiable stage along the continuum from normal aging to dementia, offering a critical window for targeted health promotion interventions. Early identification of individuals at increased risk of progression is essential for implementing preventive strategies that may delay functional decline. This study developed a transparent machine learning (ML) framework to predict diagnostic change from minor to major NCD at 12 and 24 months using baseline demographic, clinical, and multidomain neuropsychological data. **Methods**: A retrospective cohort of 162 memory clinic patients was analyzed using a rigorously controlled pipeline incorporating nested stratified cross-validation, SMOTE-based imbalance correction, and sequential forward feature selection. Logistic regression, support vector machines (SVMs), and XGBoost were evaluated, with SHapley Additive exPlanations (SHAPs) applied to ensure interpretability. **Results**: SVM achieved the most balanced predictive performance at both 12 months (accuracy = 0.90) and 24 months (accuracy = 0.81). Short-term progression was primarily driven by subtle multidomain cognitive inefficiencies, while longer-term risk reflected continued cognitive vulnerability modulated by metabolic factors such as diabetes. **Conclusions**: These findings highlight the potential of explainable ML as a health promotion tool and suggest that explainable ML can uncover clinically meaningful cognitive risk signatures at the earliest stages of NCD. By identifying modifiable systemic contributors alongside cognitive risk profiles, this framework supports precision-oriented preventive strategies and proactive longitudinal monitoring in minor NCD.

## 1. Introduction

Minor neurocognitive disorder (minor NCD), largely overlapping with mild cognitive impairment (MCI), represents a transitional and clinically heterogeneous state along the continuum from normal aging to dementia. Although often conceptualized as a prodromal stage of dementia, longitudinal evidence demonstrates that its trajectory is neither uniform nor inevitably progressive. While annual conversion rates are commonly estimated at approximately 10–15%, substantial inter-individual variability exists, with many individuals remaining stable and others progressing at markedly different rates [1,2]. Progression has been linked to baseline cognitive performance, structural neurodegeneration, molecular biomarkers, and neuropsychiatric features, yet no single predictor adequately captures outcome heterogeneity [3,4,5,6]. Even among individuals with similar baseline diagnoses, divergent longitudinal trajectories are frequently observed, underscoring the intrinsic diagnostic instability of early-stage cognitive syndromes [7].

Current theoretical frameworks conceptualize cognitive decline in minor NCD as a multidomain and dynamically evolving process shaped by the interaction between vulnerability and compensatory capacity. Domain-specific cognitive measures, particularly episodic memory and recognition processes, show high sensitivity to early decline and differential trajectories across stable versus progressive cases [8,9]. On the other hand, longitudinal evidence indicates that amnestic and multidomain subtypes follow distinct progression patterns, with multidomain impairment often associated with greater instability and higher conversion risk, particularly in the presence of vascular burden and functional decline [10]. At the same time, substantial inter-individual variability persists, highlighting the role of cognitive reserve (CR) as a moderating mechanism. Individuals with higher education or premorbid intelligence may temporarily preserve cognitive performance despite comparable biological pathology, potentially through compensatory network reconfiguration and enhanced functional connectivity in early disease stages [11,12]. However, as compensatory mechanisms diminish, trajectories may accelerate. Recent findings further identify resilience and vulnerability subgroups characterized by distinct longitudinal cognitive patterns independent of gross amyloid or metabolic load, suggesting that network-level or residual biomarkers may better capture dynamic buffering processes than static pathology measures alone [13]. Moreover, disease progression models indicate that predictive accuracy varies across time horizons, emphasizing the temporal dependency of prognostic markers [14,15,16]. These findings support a reserve-modulated, multidomain model in which heterogeneous and time-dependent diagnostic trajectories emerge from the interplay between cognitive vulnerability and adaptive capacity.

Machine learning (ML) and deep learning approaches have increasingly been applied to predict progression across the NCD continuum, leveraging multimodal neuroimaging, cognitive, genetic, and biomarker data. Recent transformer-based and multimodal fusion architectures have demonstrated strong performance in predicting NCD to dementia conversion, while integrating interpretability tools such as SHapley Additive exPlanations (SHAP) and attention maps to enhance clinical transparency [17,18]. Systematic reviews further highlight the rapid evolution of vision transformers, convolutional architectures, and hybrid multimodal frameworks, while also emphasizing persistent gaps in reproducibility and limited focus on longitudinal minor NCD progression [19,20]. Several studies report high predictive accuracy using MRI, PET, radiomics, or plasma biomarkers, particularly when combining structured clinical variables with imaging features [21,22,23,24]. However, important methodological challenges remain. Many models rely heavily on ADNI-derived datasets, raising concerns about generalizability and dataset-specific optimization [25,26]. Additionally, although cross-validation is widely implemented, external validation and robust leakage-safe pipelines are not consistently applied, limiting translational readiness [27]. While explainable AI methods such as SHAP, LIME, Grad-CAM, and counterfactual inference are increasingly incorporated to address the “black-box” problem [28,29], interpretability often remains post hoc rather than structurally embedded in model design. Τhe literature underscores the strong predictive promise of ML in NCD prognosis, yet also highlights the need for transparent, reproducible, and clinically grounded modeling frameworks that prioritize generalizability and methodological rigor [30,31,32].

Despite substantial advances in neuropsychological assessment and neuroimaging, predicting which individuals with minor NCD will exhibit diagnostic instability or progression remains a major challenge in clinical neurology. To address these gaps, the present study implemented a transparent and clinically oriented ML framework designed to model diagnostic trajectories in individuals with minor NCD. Using a robust analytic workflow (including nested cross-validation, systematic preprocessing pipelines, and sequential forward feature selection) we developed predictive models to estimate the probability of diagnostic change over 12- and 24-month intervals. All analyses were conducted in Python (version 3.10). Three classifier families (logistic regression (LR), support vector machines (SVM), and XGBoost) were evaluated to identify the most reliable approaches for prognostic stratification. Model interpretability was ensured through SHAP analyses, enabling the identification of cognitive and systemic variables that most strongly contribute to diagnostic instability. The primary objectives of this study were (1) to determine which ML approaches most accurately predict short- and medium-term diagnostic transitions in individuals with minor NCD, (2) to derive compact and clinically interpretable sets of neuropsychological and systemic predictors that may function as candidate prognostic biomarkers, and (3) to characterize the cognitive risk architecture underlying early neurodegenerative trajectories. By highlighting modifiable and clinically actionable predictors, this framework aims to support early risk stratification, improve diagnostic monitoring, and inform preventive or health-promotion strategies in populations at risk for progressive cognitive decline.

## 2. Materials and Methods

### 2.1. Study Design

This retrospective observational study analyzed structured clinical data obtained during routine neurological assessments. The study aimed to predict longitudinal diagnostic change, defined as a transition from the baseline diagnostic classification (minor NCD) to a different classification at follow-up (major NCD), at two clinically relevant time horizons: 12 months and 24 months. For the 12-month cohort, diagnostic stability (Target = 0) was more common, with 126 patients remaining stable and 36 exhibiting diagnostic change from minor NCD to major NCD. In contrast, the 24-month cohort showed a more balanced distribution, with 86 patients experiencing diagnostic change (Target = 1, i.e., major NCD) and 75 remaining stable (i.e., minor NCD). These differences in class prevalence were explicitly addressed during model development through the use of stratified sampling and SMOTE-based oversampling within the training folds.

Only baseline variables including demographic, clinical, and cognitive examination features were used as predictors, ensuring that the forecasting task relied exclusively on information available during the initial clinical encounter. All analyses adhered to established methodological guidelines for machine learning research in clinical contexts, with particular attention to preventing information leakage, preserving temporal validity, and maximizing model generalizability through nested cross-validation and standardized preprocessing procedures.

### 2.2. Participants

This study included data from 162 participants enrolled in an ongoing clinical registry maintained by the Department of Neurology at the University Hospital of Alexandroupolis. All participants had a baseline diagnosis of minor NCD and underwent structured neuropsychological evaluation as part of routine clinical assessment in the outpatient dementia clinic. Eligibility criteria required the availability of longitudinal diagnostic follow-up for a minimum period of two years following the baseline assessment. Only individuals with complete baseline clinical and cognitive data and documented diagnostic status at follow-up were included in the final analytic sample. Participants were consecutively recruited within routine clinical practice, minimizing selection bias and enhancing ecological validity. The baseline diagnostic classification was determined by experienced neurologists specializing in cognitive disorders, based on clinical examination and comprehensive neuropsychological testing. Follow-up diagnostic evaluations were conducted during routine reassessment visits at approximately 12 and 24 months. Diagnostic change was defined as a transition from the initial baseline classification to a different diagnostic category at follow-up, consistent with the operational definition described in Section 2.3.

All participants provided written informed consent prior to enrollment in the study. For individuals with dementia, consent was additionally obtained from a caregiver and/or legally authorized representative. The study protocol was approved by the Ethics Committee of the University Hospital of Alexandroupolis (Approval No. ΔΣ1/Θ68/06-04-2020). All data were processed in anonymized form.

### 2.3. Inclusion and Exclusion Criteria

All participants underwent a structured baseline clinical interview during which demographic characteristics and detailed medical history were recorded, including prior medical diagnoses, cardiovascular and metabolic conditions, neurological disorders, and history of affective diseases. As part of routine clinical evaluation in the outpatient dementia clinic, all individuals received a standardized neurological examination, comprehensive neuropsychological assessment, brain neuroimaging, and biochemical and hematological laboratory testing.

At baseline, 162 individuals fulfilled the diagnostic criteria for minor NCD according to the fifth edition of the Diagnostic and Statistical Manual of Mental Disorders (DSM-5) [33]. The diagnosis required evidence of subjective or informant-reported cognitive decline, objective cognitive impairment relative to age- and education-adjusted norms demonstrated through formal neuropsychological testing, and decline exceeding expected normal aging without meeting criteria for major neurocognitive disorder. Preservation of general cognitive functioning and independence in activities of daily living was required, and individuals with a prior diagnosis of dementia or other conditions capable of explaining cognitive impairment, such as major depression, delirium, intoxication, or psychosis, were excluded.

Additional eligibility criteria included age greater than 40 years and mild cognitive impairment defined as performance between one and one and a half standard deviations below age- and education-adjusted normative means on the Mini-Mental State Examination (MMSE) [34] and the Montreal Cognitive Assessment (MoCA) [35]. Individuals with other primary neurological diseases or those currently receiving cholinesterase inhibitors, antipsychotic medications, or anticholinergic agents were excluded. The inclusion of participants over 40 years of age allowed the identification of early cognitive vulnerability and midlife risk patterns, a period during which genetic susceptibility, vascular–metabolic burden, and lifestyle-related factors may begin to influence cognitive trajectories.

Exclusion criteria further comprised secondary causes of cognitive impairment confirmed through laboratory investigations, including vitamin B12 or folate deficiency and thyroid dysfunction, as well as structural brain abnormalities detected on conventional magnetic resonance imaging, such as territorial infarction, intracranial hemorrhage, brain tumor, hydrocephalus, or traumatic brain injury. Participants were followed prospectively with annual clinical reassessments, including repeat diagnostic evaluation at approximately 12 and 24 months.

The primary outcome of the study was progression to major neurocognitive disorder. To prevent methodological circularity, the specific neuropsychological subtests used as predictive variables in the machine learning models were not directly incorporated into the adjudication of diagnostic progression. The diagnosis of major neurocognitive disorder was established according to DSM-5 criteria and required both significant cognitive decline relative to baseline and evidence of functional impairment reflected in reduced independence in activities of daily living. Objective cognitive deterioration was operationalized as total MMSE and MoCA scores falling at least two standard deviations below age- and education-adjusted normative means. Final diagnostic determinations were made by experienced clinicians independent of the modeling process.

### 2.4. Measurements

All participants underwent a comprehensive neuropsychological assessment that included the Greek version of the Cambridge Cognitive Examination (CAMCOG) [36], administered within the framework of the Cambridge Examination for Mental Disorders of the Elderly (CAMDEX) [37]. The CAMCOG is a multidimensional cognitive screening instrument designed to evaluate a broad range of cognitive domains and is widely used in clinical and research settings for the assessment of neurocognitive disorders. The examination comprises 28 structured subtests (CAMCOG_1: time orientation; CAMCOG_2: place orientation; CAMCOG_3: comprehension-motor response; CAMCOG_4: comprehension-verbal response; CAMCOG_5: expression-naming; CAMCOG_6: expression-definitions; CAMCOG_7: comprehension-repetition; CAMCOG_8: recall; CAMCOG_9: recognition; CAMCOG_10: memory recall of remote information; CAMCOG_11: memory recall of recent information; CAMCOG_12: registration; CAMCOG_13: attention/concentration; CAMCOG_14: memory-recall; CAMCOG_15: written comprehension; CAMCOG_16: copying-drawing; CAMCOG_17: spontaneous writing; CAMCOG_18: ideational praxis; CAMCOG_19: writing under dictation; CAMCOG_20: ideomotor praxis; CAMCOG_21: tactile perception; CAMCOG_22: numerical calculations; CAMCOG_23: recall of written language; CAMCOG_24: abstract thinking; CAMCOG_25: recognition of famous people; CAMCOG_26: form stability; CAMCOG_27: function recognition; CAMCOG_28: time perception) that collectively assess orientation in time and place, language functions, attention and concentration, memory, praxis, perception, calculation, and abstract thinking. These subdomains provide a fine-grained profile of cognitive functioning across multiple domains that are commonly affected in neurodegenerative and vascular cognitive disorders. Scores are derived both at the level of individual subtests and as composite domain measures, allowing detailed characterization of cognitive strengths and weaknesses.

In the present study, individual CAMCOG subtest scores obtained at baseline were used as candidate predictive features within the machine learning framework. Rather than relying solely on global cognitive indices, the modeling approach incorporated fine-grained subdomain-level information to capture subtle multidomain patterns of impairment. This strategy enabled the identification of specific cognitive signatures associated with subsequent diagnostic change at both 12- and 24-month follow-up. Importantly, although global MMSE and MoCA scores were used for diagnostic adjudication, the machine learning models were trained primarily on CAMCOG subtest-level variables to enhance domain-specific resolution and to minimize circularity between predictor variables and diagnostic outcomes. This design allowed the predictive framework to leverage detailed neuropsychological structure while maintaining methodological rigor in outcome definition.

Demographic variables included age (in years), sex, and years of formal education. Clinical variables comprised disease duration (in years) at baseline and the presence of cardiovascular and metabolic risk factors, including hypertension, hypercholesterolemia, atrial fibrillation, and diabetes. Body mass index (BMI) [38] was evaluated for all participants and then binarized based on the cut-off score of 25. The presence of white matter lesions (WML) on neuroimaging was recorded as a binary variable. Lifestyle-related factors included smoking status and alcohol consumption. All demographic and clinical variables were collected at baseline and were incorporated as candidate predictors in the ML pipeline alongside neuropsychological measures. This combined cognitive systemic feature set enabled evaluation of the relative contribution of demographic characteristics, vascular metabolic burden, and lifestyle factors to short- and medium-term diagnostic change.

### 2.5. Machine Learning Workflow

#### 2.5.1. Data Acquisition and Preparation

The dataset consisted of structured clinical examination variables, including demographic data (age, sex, education), clinical variables (disease duration, medical history variables) and cognitive measurements. Data preparation followed a standardized protocol involving removal of duplicate entries, coercion of mixed-format variables into numeric types, and verification of consistency across patient records. Two clinical experts reviewed all candidate predictors to identify and remove variables that could directly or indirectly reveal the diagnostic outcome, thereby preventing information leakage. After preprocessing and expert validation, the analytic dataset consisted of a curated set of neurological examination variables and a binary outcome indicating whether the diagnosis changed (1) or remained stable (0) at each follow-up interval. Diagnostic stability refers to the baseline diagnosis of minor NCD which remained stable across visits whereas diagnostic instability refers to transition from minor NCD to major NCD.

#### 2.5.2. Data Splitting, Preprocessing, and Validation

All analyses were conducted in Python (version 3.10, accessed on 18 January 2026). Stratified sampling was used throughout all stages of the analysis to preserve class proportions. Model development followed a nested stratified five-fold cross-validation procedure to obtain unbiased performance estimates while ensuring complete isolation of training and validation data. All preprocessing steps were performed exclusively within the training folds to avoid information leakage. Numerical variables were standardized using z-score normalization, and class imbalance was addressed using the Synthetic Minority Oversampling Technique (SMOTE), applied only to the training partitions. These transformations were implemented as a unified pipeline so that scaling, oversampling, hyperparameter optimization, and model fitting were reproduced identically in every fold. Hyperparameters were tuned within the inner cross-validation loop using grid search with accuracy as the scoring metric. The best-performing configuration was then evaluated in the outer loop. After nested cross-validation, each optimal model was refitted on the complete training–validation dataset and evaluated once on the independent test set. Given the relatively modest sample size in relation to the complexity of the modeling pipeline, particular care was taken to minimize overfitting through strict separation of training and testing procedures and the use of nested cross-validation. To further clarify the validation strategy, nested cross-validation was used exclusively within the training data to perform hyperparameter tuning and feature selection, ensuring unbiased internal performance estimation. Following this procedure, the optimal model configuration was refitted on the full training–validation dataset and subsequently evaluated on a held-out independent test set. This approach ensured a clear separation between model selection and final performance assessment.

#### 2.5.3. Feature Selection

Feature selection was conducted using a wrapper-based Sequential Forward Selection (SFS) procedure applied independently to each classifier. Starting from an empty feature set, the algorithm tested all possible one-feature extensions of the current subset and evaluated each candidate using a full preprocessing and modeling pipeline. For every candidate feature set, hyperparameters were tuned via an embedded grid search, and classification accuracy was estimated using five-fold cross-validation. The feature whose inclusion produced the largest improvement in accuracy was added to the selected subset, and the procedure was repeated until all variables had been ranked. This approach generated a performance trajectory across successive subset sizes and produced a classifier-specific ranking of features based on their incremental contribution to predictive performance. The subset yielding the highest mean accuracy during cross-validation was selected for final model evaluation.

#### 2.5.4. Machine Learning Algorithms

Three supervised ML classifiers were evaluated using the unified cross-validated pipeline: logistic regression (LR), support vector machines (SVMs), and XGBoost. Logistic regression served as an interpretable linear baseline model, with penalty type and regularization strength tuned through grid search. The SVM models incorporated both linear and radial-basis-function (RBF) kernels. During hyperparameter tuning, the pipeline jointly evaluated linear SVMs and RBF-kernel SVMs by exploring model families in parallel: the linear SVM varied the regularization parameter *C*, whereas the RBF SVM varied both *C* and the kernel bandwidth *γ*. This approach allowed the cross-validation procedure to determine whether a linear or nonlinear decision boundary provided the best representation of the clinical feature space. The XGBoost classifier was optimized using grid search across the number of trees, tree depth, learning rate, and minimum child weight. For all classifiers, model performance was quantified using accuracy, precision, recall, F1-score, ROC-AUC, and the mean normalized confusion matrix averaged across the outer cross-validation folds.

#### 2.5.5. Model Interpretation

Model interpretability was assessed using SHAPs, which quantify the marginal impact of each predictor on a model’s output. SHAP analysis was conducted only for the best-performing classifier as determined by nested cross-validation. The appropriate SHAP explainer was chosen according to the model architecture, and SHAP values were computed using the final selected feature subset. Summary SHAP plots were generated to identify the most influential predictors and to visualize how variations in clinical variables affected the estimated probability of diagnostic change from minor NCD to major NCD. This interpretability analysis provided transparent insight into the drivers of the model’s predictions and supported the identification of clinically meaningful examination features.

## 3. Results

### 3.1. Demographic and Clinical Characteristics of the Group

The total group included 162 participants (49 males, 113 females) with baseline diagnosis of minor NCD. Mean age was 70.17 years (SD = 7.53 years) while mean education was 8.43 years (SD = 4.91 years). Mean disease duration of minor NCD was 1.65 years (SD = 1.25 years). Regarding clinical factors, 35.2% of the participants had hypertension (N = 57/162), 30.2% had hypercholesterolemia (N = 49/162), 10.5% had atrial fibrillation (N = 17/162), and 14.2% had diabetes (N = 23/162). In the total group, 35.8% of the participants had white matter lesions (N = 58/162). Regarding obesity, 14.8% of the participants had a BMI > 25 (N = 24/162). A current smoking history was reported in 11.7% of the participants (N = 19/162) whereas only 1.2% of the participants reported increased alcohol consumption (N = 2/162).

### 3.2. Prediction of Diagnostic Change at 12 Months

Three ML classifiers were trained and evaluated using the unified analytical pipeline described in the Methods section. Model performance was assessed for two clinically relevant prediction horizons: diagnostic change at 12 months and diagnostic change at 24 months. Evaluation was conducted using nested stratified cross-validation, with performance metrics reported on held-out test data. Across both prediction tasks, substantial variability in performance was observed across algorithms, and the embedded feature-selection procedures yielded distinct, model-specific subsets of predictive variables, reflecting differences in model complexity and decision structure.

#### 3.2.1. Prediction of Diagnostic Change at 12 Months

For the 12-month diagnostic change prediction task, LR, SVM, and XGBoost were evaluated using SFFS embedded within a nested cross-validation framework. Model performance was assessed using accuracy, precision, recall, F1-score, and ROC–AUC (Table 1).

Among the evaluated classifiers, SVM achieved the highest overall accuracy (0.9011) and the highest F1-score (0.7576), indicating strong overall classification performance and balance between precision and recall. SVM achieved the highest overall accuracy (0.9011) and the highest F1-score (0.7576), indicating strong overall classification performance, although recall remained moderate (0.7179), suggesting that a proportion of progressive cases were not detected. Logistic Regression exhibited slightly lower accuracy (0.8576) but achieved a competitive ROC–AUC (0.8640) and a balanced trade-off between precision (0.6929) and recall (0.7500), reflecting stable discrimination performance. Importantly, LR required a larger feature subset (14 features), indicating greater reliance on multiple predictors to achieve comparable performance. In contrast, XGBoost showed similar accuracy to LR (0.8580) and high precision (0.8033) but suffered from substantially reduced recall (0.4786) and the lowest ROC–AUC (0.7565) among the models, suggesting limited sensitivity and weaker discriminative capability despite using the smallest feature set (8 features). Considering the clinical importance of balanced sensitivity and discrimination, SVM was selected as the most suitable model among the evaluated classifiers, although differences in performance across models were relatively modest. SVM was identified as the most suitable model for the 12-month prediction horizon, offering the best compromise between performance metrics while maintaining a relatively compact feature subset (11 features). The optimal SVM hyperparameters were identified as C = 2, gamma = ‘auto’, and kernel = ‘rbf’.

**Figure 1:**
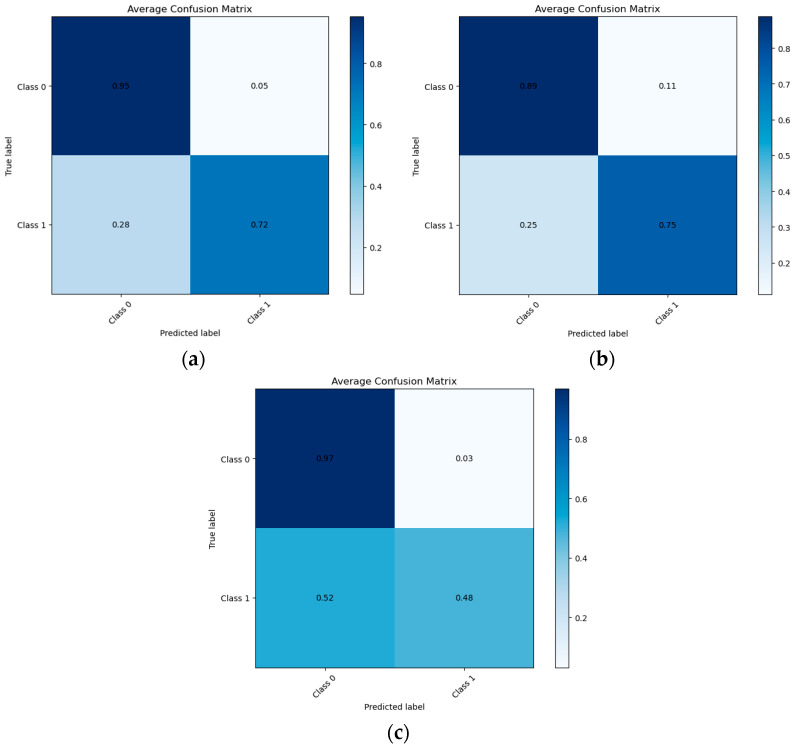
Normalized Confusion Matrix for the employed classifiers, (**a**) SVM, (**b**) LR and (**c**) XGBoost at 12 months.

Figure 1 presents the normalized confusion matrix of the employed classifiers. The SVM model demonstrates high specificity, correctly identifying 95% of individuals without diagnostic change, while achieving acceptable sensitivity, correctly detecting 72% of individuals who experienced diagnostic progression within 12 months. Although a proportion of progressive cases were misclassified, the overall pattern indicates that the model effectively distinguishes clinically stable from deteriorating patients, supporting its potential utility in early risk stratification.

SVM selected a compact subset of 11 predictors, most of which were derived from CAMCOG subtests, alongside demographic and clinical history variables. These features captured multidomain cognitive performance, comorbidities, and baseline diagnostic assessment, suggesting that early neurocognitive status plays a central role in short-term diagnostic instability.

These findings indicate that multidomain cognitive test performance, alongside vascular and metabolic risk factors, are the strongest predictors of diagnostic instability at 12 months. The final interpretation of the SVM model using SHAP values will be presented in the subsequent section.

#### 3.2.2. Best Performing Classifier for the 12-Month Diagnostic Change

**Figure 2:**
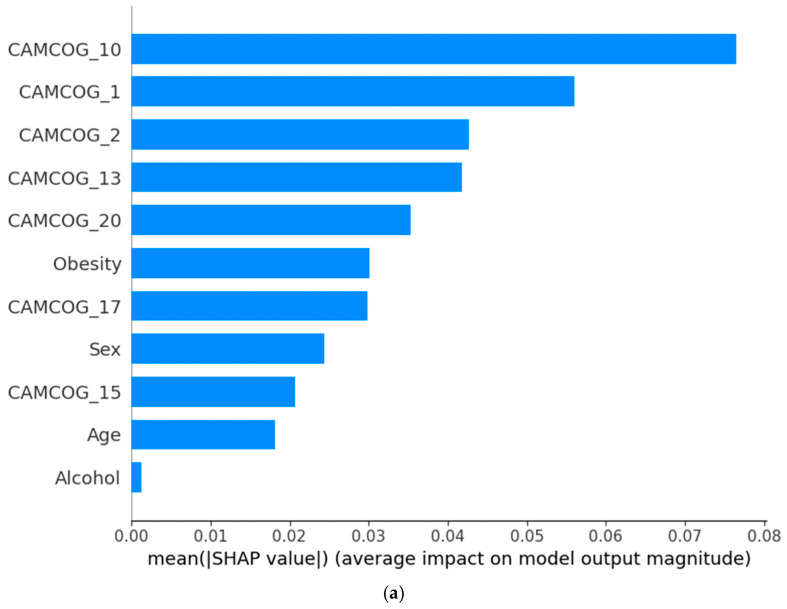

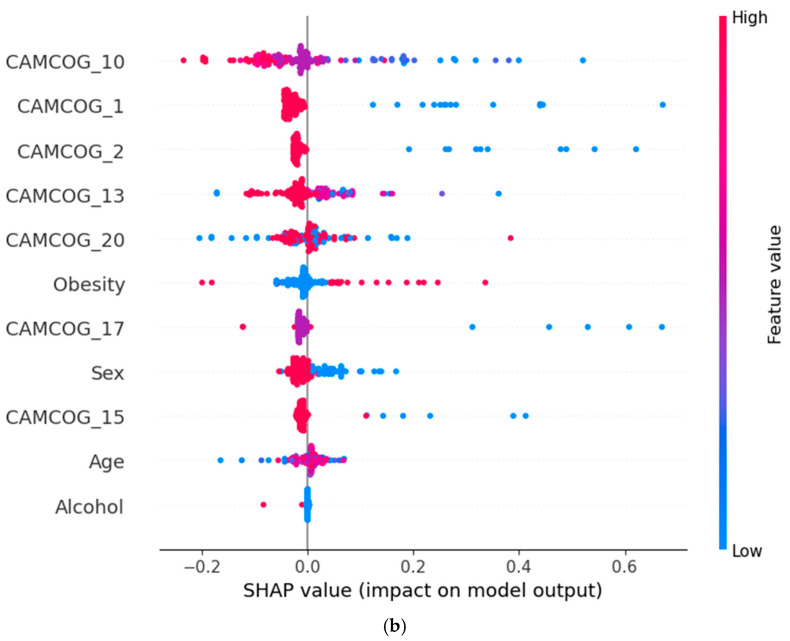
(**a**) Bar plot of mean absolute SHAP values ranking the most influential features and (**b**) SHAP summary plot illustrating the direction and magnitude of feature contributions to 12-month diagnostic change predictions generated by the SVM model. (For a detailed explanation of CAMCOG variables, see Table 2).

Figure 2 presents the SHAP-based interpretation of the SVM model, highlighting the most influential predictors of 12-month diagnostic change. Features are ranked according to their mean absolute SHAP values (Figure 2a), while the SHAP summary plot (Figure 2b) illustrates the direction and magnitude of each feature’s contribution at the individual patient level. Each point represents a single participant, with positive SHAP values indicating an increased predicted probability of diagnostic change and negative values indicating a protective effect. Feature values are color-coded, with red denoting higher values and blue lower values.

Performance on specific CAMCOG subtests emerged as the dominant drivers of model predictions. In particular, CAMCOG_10, CAMCOG_1, CAMCOG_2, and CAMCOG_13 showed the highest contributions. Higher scores on these cognitive measures were consistently associated with negative SHAP values, indicating a reduced likelihood of diagnostic progression, whereas lower scores shifted predictions toward an increased probability of change. This pattern underscores the central role of multidomain cognitive performance in early identification of individuals at risk of clinical deterioration.

Beyond cognitive measures, obesity and sex contributed modestly to the model’s predictions, suggesting an additional but secondary influence of demographic and metabolic factors. Age demonstrated a smaller effect size, while alcohol consumption showed minimal impact on prediction outcomes. Overall, the SHAP patterns indicate that the SVM model primarily relies on fine-grained cognitive performance metrics, with limited contribution from non-cognitive variables.

Collectively, these findings suggest that subtle deficits across specific cognitive domains, rather than global demographic or lifestyle factors, form the core explanatory structure of the SVM model for predicting 12-month diagnostic stability or progression. This supports the clinical relevance of detailed cognitive profiling in short-term prognostic assessment.

**Table 2:** Selected Features for the Optimal Logistic Regression Model.

Selected Feature	Description
CAMCOG_2	Place orientation score (baseline)
CAMCOG_1	Time orientation score (baseline)
CAMCOG_10	Memory recall of remote information score (baseline)
CAMCOG_17	Spontaneous writing score (baseline)
Age	Age (in years)
Obesity	Presence (BMI ≥ 25); Absence (BMI < 25)
Alcohol	Presence; Absence
CAMCOG_13	Attention/Concentration score (baseline)
CAMCOG_15	Written comprehension score (baseline)
CAMCOG_20	Ideomotor praxis score (baseline)
Sex	Sex (Male; Female)

#### 3.2.3. Prediction of Diagnostic Change at 24 Months

For the 24-month diagnostic change prediction task, LR, SVM, and XGBoost were evaluated using nested cross-validation combined with SFFS. Model performance was assessed using accuracy, ROC–AUC, precision, recall, and F1-score (Table 3).

Across all evaluated metrics, the SVM demonstrated the most balanced overall performance among the evaluated models. Specifically, SVM achieved the highest accuracy (0.8083) and the highest F1-score (0.8183), reflecting an optimal balance between precision (0.8360) and recall (0.8039). This indicates robust sensitivity to diagnostic change while maintaining reliable positive predictions. Logistic Regression showed competitive performance, achieving the highest ROC–AUC (0.8269) and high precision (0.8558), suggesting strong discriminative capability. However, its overall accuracy (0.7964) and F1-score (0.7995) were lower than those achieved by SVM, indicating a less favorable balance between sensitivity and overall classification performance. In contrast, XGBoost utilized a substantially larger feature set (21 features) but did not translate this increased complexity into superior performance. Although precision (0.8231) and recall (0.7725) were acceptable, XGBoost achieved the lowest accuracy (0.7843) and a comparatively lower ROC–AUC (0.8181), suggesting reduced discriminative efficiency.

Considering overall accuracy, sensitivity, and balanced predictive performance (key factors for long-term clinical prognosis) the SVM classifier was identified as the most suitable model within the current experimental setting for predicting diagnostic change at 24 months. The best-performing SVM configuration employed the following hyperparameters: C = 2, gamma = ‘auto’, and kernel = ‘rbf’.

**Figure 3:**
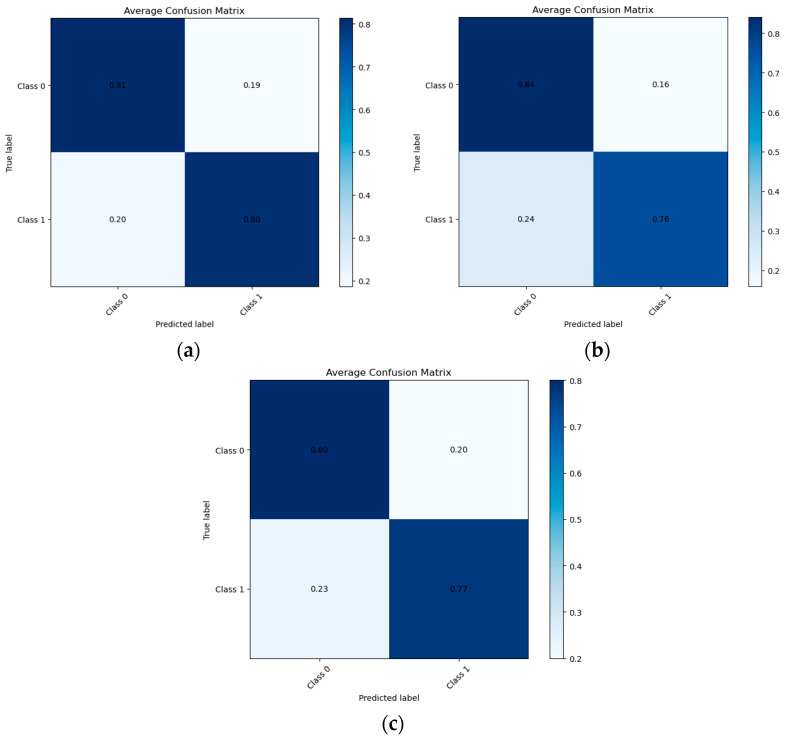
Normalized Confusion Matrix for the employed classifiers, (**a**) SVM, (**b**) LR and (**c**) XGBoost at 24 months.

Figure 3 presents the normalized confusion matrix of the SVM, LR and XGBoost classifier for the 24-month prediction horizon. The SVM model correctly identified 81% of individuals without diagnostic change, demonstrating good specificity, while 80% of individuals with diagnostic progression were correctly classified, indicating strong sensitivity. Misclassification rates were comparable across classes (19% for Class 0 and 20% for Class 1), suggesting balanced performance. Overall, the confusion matrix supports the clinical utility of the SVM model in longer-term risk stratification, with consistent discrimination between stable and progressive diagnostic states.

The best-performing SVM model selected ten predictors. These features spanned multiple CAMCOG cognitive domains, and metabolic status. This selection pattern reflects the increasing contribution of systemic health variables to longer-term diagnostic trajectories, in contrast to the more cognitively dominated patterns observed at earlier time points.

These findings suggest that cognitive performance, when combined with systemic health indicators such as cardiovascular and metabolic comorbidities, provides a strong basis for predicting diagnostic stability over a 24-month period. The predominance of CAMCOG subdomains among the selected features underscores the central role of detailed neurocognitive assessment, while the additional inclusion of vascular and lifestyle factors suggests a cumulative influence of systemic health on longer-term diagnostic change.

#### 3.2.4. Best Performing Classifier for the 24-Month Diagnostic Change

**Figure 4:**
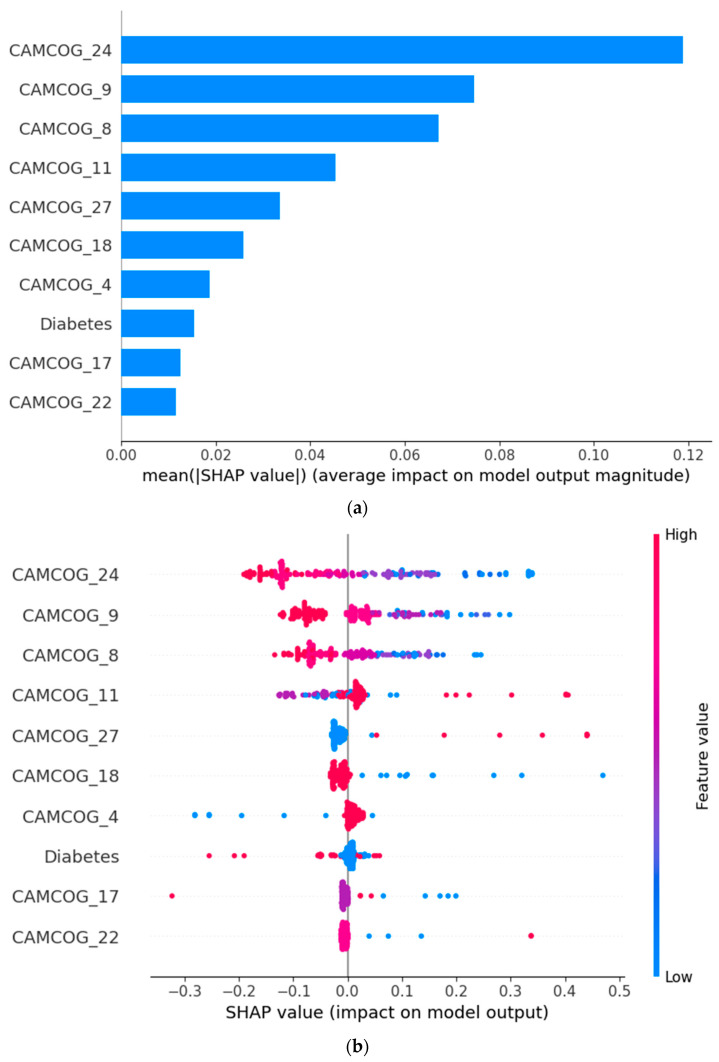
(**a**) Bar plot of mean absolute SHAP values ranking the most influential features and (**b**) SHAP summary plot illustrating the direction and magnitude of feature contributions to 24-month diagnostic change predictions generated by the SVM model. (For a detailed explanation of CAMCOG variables, see Table 4).

Figure 4 presents the SHAP-based interpretation of the SVM model for the 24-month diagnostic change prediction task. Features are ranked according to their mean absolute SHAP values (Figure 4a), while the SHAP summary plot (Figure 4b) illustrates the direction and magnitude of each feature’s contribution at the individual patient level. Each point corresponds to a single participant, with positive SHAP values indicating an increased predicted probability of diagnostic change and negative values indicating a protective effect. Feature values are color-coded, with red representing higher values and blue lower values.

Several CAMCOG subtests emerged as the most influential predictors of long-term diagnostic change, particularly CAMCOG_24, CAMCOG_9, CAMCOG_8, and CAMCOG_11. Higher performance on these cognitive measures was predominantly associated with negative SHAP values, indicating a reduced likelihood of diagnostic progression over 24 months. Conversely, lower cognitive scores consistently shifted model predictions toward an increased risk of diagnostic instability, highlighting the importance of preserved multidomain cognitive functioning in long-term prognosis.

Additional CAMCOG components, including CAMCOG_27, CAMCOG_18, CAMCOG_4, CAMCOG_17, and CAMCOG_22, contributed more modestly but consistently to model predictions, suggesting that subtle deficits across multiple cognitive domains cumulatively influence long-term diagnostic trajectories.

Among non-cognitive factors, diabetes showed a measurable contribution, with its presence generally associated with a shift toward higher predicted risk of diagnostic change. This finding supports the role of metabolic comorbidity as an important modifier of long-term cognitive and diagnostic outcomes.

Overall, the SHAP distributions indicate that the SVM model’s assessment of 24-month diagnostic change risk is driven primarily by fine-grained cognitive performance across multiple CAMCOG subdomains, with metabolic health factors providing secondary but clinically relevant influence. This combined cognitive–systemic profile underscores the multifactorial nature of long-term diagnostic progression.

**Table 4:** Selected Features for the Optimal SVM Model (24-Month Prediction).

Selected Feature	Description
CAMCOG_8	Recall score (baseline)
CAMCOG_27	Function recognition score (baseline)
CAMCOG_24	Abstract thinking score (baseline)
CAMCOG_22	Numerical calculations score (baseline)
CAMCOG_9	Recognition score (baseline)
CAMCOG_18	Ideational praxis score (baseline)
CAMCOG_11	Memory recall of recent information score (baseline)
Diabetes	Presence; Absence
CAMCOG_4	Comprehension-verbal response score (baseline)
CAMCOG_17	Spontaneous writing score (baseline)

## 4. Discussion

The present study suggests that explainable ML models can provide meaningful predictive insight into short- and medium-term diagnostic change in individuals with minor NCD using only baseline clinical and neuropsychological data. Across both prediction horizons, the SVM classifier consistently showed the most balanced performance among the evaluated models, outperforming logistic regression and XGBoost in overall accuracy and F1-score while maintaining strong discriminative capacity. Importantly, model interpretability analyses revealed distinct yet convergent explanatory patterns across time. At the 12-month horizon, predictive performance was driven predominantly by fine-grained multidomain cognitive measures derived from CAMCOG subtests, suggesting that subtle baseline cognitive inefficiencies represent the primary determinants of early diagnostic instability. In contrast, prediction at 24 months reflected a broader explanatory structure in which cognitive variables remained central but were complemented by metabolic factors, most notably diabetes, indicating a progressively increasing contribution of systemic health burden over time. These findings suggest that diagnostic change in minor NCD follows a temporally dynamic pattern in which early progression is primarily shaped by multidomain cognitive vulnerability, whereas longer-term trajectories increasingly reflect the interaction between baseline cognitive deficits and systemic metabolic risk. The integration of transparent ML approaches within clinical workflows may therefore enable early, individualized risk stratification while preserving interpretability and clinical relevance. Importantly, the predictive associations identified by the present machine learning models should not be interpreted as evidence of causal or etiological mechanisms. The contribution of each feature, as quantified through SHAP values, reflects its role within the model’s predictive structure rather than its direct involvement in disease pathophysiology. Therefore, the present findings should be understood within a prognostic modeling framework, aimed at risk estimation, rather than causal inference.

### 4.1. Interpretation of Short-Term Prediction (12-Month Horizon)

The 12-month findings indicate that early diagnostic change is primarily driven by fine-grained multidomain cognitive vulnerability, rather than by demographic or lifestyle factors. In the SHAP analysis of the optimal RBF-SVM model, the most influential predictors were orientation in time, orientation in place, memory recall and attention/concentration, with lower scores consistently increasing predicted risk of diagnostic instability. This pattern is consistent with prior literature showing that orientation and memory subscores carry prognostic value in early disease stages. Using ADNI data, Choe et al. demonstrated that MMSE orientation (time-related items) and delayed recall were inversely associated with conversion to Alzheimer’s disease, suggesting that subtle disorientation and memory inefficiency may signal early progression risk [39]. Similarly, Thabtah and Peebles identified delayed recall as a leading early marker in ML models of MCI progression, with orientation-related measures emerging as important features in stage discrimination [40]. Longitudinal CAMCOG-based evidence further supports the short-term sensitivity of these domains, as Tsiakiri et al. observed measurable decline in orientation and memory over six months in individuals with minor NCD [41]. The contribution of attention/concentration supports a multidomain interpretation. Domain-level early impairments in attention and calculation have been reported in systemic-risk cohorts by Levassort et al., suggesting that attentional control may interact with memory processes in early vulnerability states [42]. Together, these findings align with a framework of subclinical executive–memory interactions, where modest deficits across interconnected domains cumulatively increase short-term instability risk, namely change from minor NCD to major NCD. On the other hand, age and alcohol consumption showed minimal contribution in our model. This likely reflects the short prediction horizon: while demographic and lifestyle exposures may influence long-term trajectories, proximal neurocognitive status appears more decisive within a one-year window.

From a methodological point of view, the superior performance of the nonlinear RBF-SVM over logistic regression suggests that early progression is not governed by simple additive effects. Instead, the risk boundary may be nonlinear, emerging from interactions between orientation, memory, and attention systems. This interpretation is consistent with ML-based progression studies emphasizing domain interdependencies and stage-dependent feature salience [40]. The 12-month results support a model in which early diagnostic change reflects multidomain micro-level cognitive inefficiency shaped by nonlinear domain interactions, consistent with the broader MCI-to-dementia transition literature.

An important methodological consideration is whether the observed predictive performance reflects true longitudinal risk estimation or simply the identification of individuals already close to the diagnostic threshold at baseline. In the present study, this risk was mitigated by ensuring that the neuropsychological features used as predictors (CAMCOG subtests) were not directly incorporated into diagnostic adjudication, which relied on global cognitive indices (MMSE, MoCA) and functional criteria. Furthermore, the model consistently selected multidomain cognitive patterns rather than single global severity indicators, suggesting that it captures distributed cognitive vulnerability rather than threshold proximity alone. Nevertheless, some degree of overlap between baseline severity and progression risk cannot be entirely excluded and represents an inherent limitation of clinical prognostic modeling.

### 4.2. Interpretation of Medium-Term Prediction (24-Month Horizon)

At the 24-month horizon, predictive performance remained primarily anchored in multidomain cognitive measures, particularly recall, recognition, and abstract thinking, indicating that episodic memory and higher-order executive processes continue to form the core substrate of diagnostic instability, namely change from minor NCD to major NCD. However, in contrast to the 12-month model, systemic variables such as diabetes entered the selected feature set, albeit with more modest explanatory weight. This pattern suggests not a replacement of cognitive drivers, but a transition from a predominantly cognitive model to a cognitive–systemic interaction framework. The dominant contribution of recall and recognition is consistent with evidence that episodic memory systems are central to progression along the NCDs continuum, particularly when multidomain involvement is present [43]. Abstract thinking, reflecting executive–frontal functioning, further supports the idea that long-term progression involves distributed network vulnerability rather than isolated hippocampal dysfunction [44,45].

Although diabetes did not emerge as a primary driver, its inclusion in the optimal feature subset suggests a secondary modulatory role. This is consistent with models proposing that vascular and metabolic comorbidities exert gradual effects on structural connectivity and white matter integrity, thereby influencing cognitive trajectories over time [46,47]. Importantly, such systemic burden may not produce immediate short-term instability but may interact with pre-existing cognitive weakness, becoming more detectable as follow-up lengthens. Within a cognitive reserve depletion framework, individuals with subtle baseline multidomain inefficiencies may initially compensate; however, as compensatory capacity diminishes, even moderate systemic stressors can amplify vulnerability. This interactional perspective aligns with multifactorial models of sporadic neurodegeneration that position metabolic factors as contributors rather than sole determinants of progression [48].

The 24-month findings suggest that medium-term diagnostic change reflects baseline multidomain cognitive vulnerability modulated by systemic health burden rather than dominated by the burden. In this view, cognition remains the structural core of risk, while vascular–metabolic factors gradually shape trajectory expression over time.

The superior performance of the RBF-SVM model may further reflect its ability to capture nonlinear relationships among cognitive domains. Unlike linear models, SVM with a radial basis function kernel can model complex interactions between features, which is particularly relevant in the context of multidomain cognitive decline where deficits across domains may interact in a non-additive manner. This supports the interpretation that early diagnostic instability may arise from interconnected domain-level vulnerabilities rather than isolated impairments.

### 4.3. Conceptual Model: Cognitive Vulnerability with Gradual Modulation

The comparison between 12- and 24-month prediction horizons suggests that diagnostic change in minor NCDs reflects a time-dependent and heterogeneous process, rather than a fixed trajectory. Across both intervals, multidomain cognitive performance remained central, yet longitudinal modeling studies indicate that early progression follows diverse and nonlinear pathways. Hybrid event-based and network-diffusion approaches demonstrate that pathological spread patterns vary substantially at early stages and tend to converge over time, supporting a dynamic model of disease evolution [49]. Similarly, multi-axis disentanglement frameworks reveal multiple latent disease trajectories within minor NCD, challenging single-path progression assumptions [50]. ML studies further confirm that distinguishing stable from progressive NCDs requires modeling heterogeneous conversion patterns [51,52,53]. Our findings are consistent with a framework in which baseline multidomain cognitive vulnerability constitutes the primary substrate of progression, while systemic and lifestyle factors gradually modulate trajectory expression over time. Rather than acting as independent drivers, these modifiers interact with an already vulnerable cognitive system. The concept of CR provides a central explanatory axis. Reiter et al. showed that CR relates to brain volumetrics differently across MCI subtypes, indicating stage- and phenotype-dependent buffering effects [54]. Education has also been identified as protective against NCDs and advanced brain aging [55,56]. At the neural level, preserved multiscale network complexity and long-distance interactions support higher cognitive function [57], while large ADNI analyses emphasize resilience and heterogeneity in disease course [58]. Together, these findings support a reserve-based buffering model in early decline.

Vascular, metabolic, and psychological factors contribute to cognitive variability but appear to operate interactively. Structural equation modeling shows that metabolic/vascular risk and depression influence executive attention and memory domains [59]. Hypertension and stroke are associated with MCI risk [60], while real-world cohorts demonstrate that multimorbidity significantly shapes clinical presentation [61,62]. Genetic and biological modifiers further interact with lifestyle exposures. APOE ε4 modifies the effect of sleep on decline [63] and strengthens the predictive value of olfactory impairment [64]. Sex-specific modulation of progression has also been documented [65,66]. Molecular evidence indicates that amyloid deposition precedes symptoms and relates to future decline [67], while tau imaging and biomarker frameworks highlight stage-dependent clinical utility [58,68]. On the other hand, lifestyle and biological exposures appear capable of influencing network integrity and biomarker expression. Physical activity and sleep relate to hippocampal microstructural integrity independent of amyloid burden [69], and dietary intervention (EVOO) modulates AD hallmarks and oxidative stress markers in MCI [70]. These findings support a vascular–metabolic acceleration hypothesis, whereby systemic exposures gradually amplify baseline vulnerability rather than initiate decline.

### 4.4. Methodological Contribution and Limitations

A key contribution of this study is the implementation of a rigorously controlled and fully interpretable ML framework. The integration of nested stratified cross-validation, SMOTE restricted to training folds, and wrapper-based sequential forward feature selection minimized information leakage and overfitting, common limitations in prognostic modeling within NCDs research. The use of SHAP-based interpretability enabled quantification of relative feature importance, demonstrating the dominance of multidomain cognitive variables while preventing overinterpretation of secondary predictors. This is particularly relevant given the heterogeneity of NCDs populations and variability in risk factor expression across biological and clinical subgroups. Finally, the superior performance of the nonlinear SVM underscores the need to model non-additive interactions among cognitive domains, aligning with contemporary multidimensional disease frameworks. The pipeline offers a transparent and replicable template for precision prognostic modeling in memory clinic settings.

This study has several limitations. It was conducted in a single memory clinic using a retrospective design and a relatively modest sample size, which may limit generalizability to broader populations. Given the relatively modest sample size and the complexity of the modeling pipeline (including feature selection, hyperparameter tuning, and oversampling), performance estimates should be interpreted with caution, as they may be subject to a degree of optimism despite the use of nested cross-validation. Another important consideration concerns the generalizability of the proposed model. Although nested cross-validation provides robust internal validation and reduces the risk of overfitting, the study is based on a single-center cohort with specific demographic and clinical characteristics. As a result, the extent to which the model can be directly transferred to other populations or clinical settings remains uncertain. The use of structured clinical and neuropsychological variables may support broader applicability compared to highly specialized imaging-based models; however, external validation in independent cohorts is essential to confirm transportability and calibration stability. Although nested cross-validation and strict separation of training and testing procedures were applied to minimize overfitting, the use of SMOTE to address class imbalance may introduce synthetic patterns that differ from real-world distributions. The use of SMOTE to address class imbalance represents both a methodological strength and a potential limitation. While SMOTE improves the representation of the minority class and may enhance model sensitivity, it generates synthetic samples based on existing data patterns, which may not fully capture the complexity of real-world clinical variability, particularly in small datasets. Although SMOTE was applied strictly within training folds to prevent information leakage, its use may still contribute to optimistic performance estimates. Future studies using larger and more diverse cohorts are needed to validate model performance without reliance on synthetic oversampling. In addition, the models relied exclusively on baseline variables, without incorporating quantitative neuroimaging, molecular biomarkers, or longitudinal metabolic measures, potentially underestimating biological contributors to progression. Systemic variables were encoded in a simplified binary form, limiting assessment of severity effects. External validation in independent and more diverse cohorts is therefore necessary to confirm robustness, calibration stability, and clinical transportability of the proposed framework. In addition to conventional performance metrics, future work may further emphasize precision–recall-based evaluation frameworks, which are particularly informative in imbalanced clinical prediction settings where accurate identification of minority classes (e.g., progression cases) is critical.

### 4.5. Clinical Implications

The findings highlight the importance of fine-grained multidomain neuropsychological assessment for early risk stratification in minor NCD. Subtest-level measures provided stronger prognostic signal than broad demographic or lifestyle factors, emphasizing the value of detailed baseline profiling. The temporal differentiation between short- and medium-term prediction supports a dynamic monitoring approach, where cognition remains central and systemic factors inform longer-term follow-up strategies. Importantly, SHAP-based interpretability enhances clinical applicability by clarifying which domains drive individual risk, facilitating precision-oriented decision support within heterogeneous NCDs populations.

While it is well established that multidomain cognitive impairment is associated with increased risk of progression, the present study extends this knowledge by providing a quantitative and time-sensitive modeling framework. Rather than confirming a general clinical observation, the proposed approach formalizes this relationship into a predictive structure, identifies compact and domain-specific feature subsets, and demonstrates that the relative contribution of cognitive and systemic factors varies across different temporal horizons. The integration of explainable machine learning further enables individualized risk profiling, moving beyond group-level associations toward precision-oriented prognostic assessment.

## 5. Conclusions

This study provides preliminary evidence that diagnostic transitions in minor NCD are not random or solely time-dependent but may reflect a structured and dynamically evolving pattern of multidomain cognitive vulnerability that can already be detected at the initial clinical assessment. By integrating rigorous methodological safeguards with nonlinear modeling and transparent SHAP-based interpretability, we show that subtle baseline inefficiencies across specific cognitive domains contain meaningful prognostic information, while systemic factors appear to function as gradual modulators of risk rather than primary drivers of diagnostic change. Importantly, the identified predictors form compact and clinically interpretable profiles that may serve as candidate prognostic markers of early cognitive decline. These results support a shift beyond static cross-sectional assessment toward a trajectory-based framework for evaluating individuals with minor NCD. Within this perspective, explainable ML can complement clinical expertise by highlighting latent cognitive risk signatures and supporting individualized prognostic stratification. With further external validation, such approaches may contribute to earlier identification of patients at risk for progressive neurodegenerative disorders, facilitate more precise longitudinal monitoring, and inform targeted preventive and health-promotion strategies within clinical neurology.

## Figures and Tables

**Table 1:** Performance of Machine Learning Models for 12-Month Diagnostic Change Prediction.

Model	Accuracy	ROC–AUC	Precision	Recall	F1-Score	Selected Features
LR	0.8576	0.8640	0.6929	0.7500	0.7149	14
SVM	0.9011	0.8560	0.8350	0.7179	0.7576	11
XGBoost	0.8580	0.7565	0.8033	0.4786	0.5939	8

Notes. LR = Logistic Regression; SVM = Support Vector Machine.

**Table 3:** Performance of ML Models for 24-Month Diagnostic Change Prediction.

Model	Accuracy	ROC–AUC	Precision	Recall	F1-Score	Selected Features
LR	0.7964	0.8269	0.8558	0.7601	0.7995	8
SVM	0.8083	0.8223	0.8360	0.8039	0.8183	10
XGBoost	0.7843	0.8181	0.8231	0.7725	0.7919	21

Notes. LR = Logistic Regression; SVM = Support Vector Machine.

## Data Availability

Data are available upon request by the authors. The original contributions presented in this study are included in the article. Further inquiries can be directed to the corresponding author(s).
